# A Continuous Blood Pressure Estimation Method Using Photoplethysmography by GRNN-Based Model

**DOI:** 10.3390/s21217207

**Published:** 2021-10-29

**Authors:** Zheming Li, Wei He

**Affiliations:** State Key Laboratory of Power Transmission Equipment and System Security and New Technology, Chongqing University, Chongqing 400044, China; lzm708@cqu.edu.cn

**Keywords:** blood pressure waveform, photoplethysmogram, neural network, blood pressure estimation, harmonic

## Abstract

Compared with diastolic blood pressure (DBP) and systolic blood pressure (SBP), the blood pressure (BP) waveform contains richer physiological information that can be used for disease diagnosis. However, most models based on photoplethysmogram (PPG) signals can only estimate SBP and DBP and are susceptible to noise signals. We focus on estimating the BP waveform rather than discrete BP values. We propose a model based on a generalized regression neural network to estimate the BP waveform, SBP and DBP. This model takes the raw PPG signal as input and BP waveform as output. The SBP and DBP are extracted from the estimated BP waveform. In addition, the model contains encoders and decoders, and their role is to be responsible for the conversion between the time domain and frequency domain of the waveform. The prediction results of our model show that the mean absolute error is 3.96 ± 5.36 mmHg for SBP and 2.39 ± 3.28 mmHg for DBP, the root mean square error is 5.54 for SBP and 3.45 for DBP. These results fulfill the Association for the Advancement of Medical Instrumentation (AAMI) standard and obtain grade A according to the British Hypertension Society (BHS) standard. The results show that the proposed model can effectively estimate the BP waveform only using the raw PPG signal.

## 1. Introduction

Blood pressure (BP) is an important physiological index for diagnosing diseases, observing changes in the condition and judging the effect of treatment. There are many cardiovascular diseases that can increase or decrease blood pressure, such as atherosclerosis [1], Renal Artery Stenosis [2], chronic malnutrition [3], and mitral valve stenosis [4]. Raised blood pressure is known as hypertension, and reduced blood pressure is known as hypotension. Many investigations in arterial hemodynamics have indicated that the human blood pressure waveform contains more information than diastolic blood pressure (DBP) and systolic blood pressure (SBP), and this information includes indices describing left ventricular systolic function and arterial properties [5]. Therefore, it is necessary to measure or estimate SBP, DBP and BP waveform (continuous BP) at the same time.

Sphygmomanometers are currently widely used BP measuring instruments. These instruments measure BP via an inflatable cuff across the arm of a patient and BP is determined at the height of the mercury column [6]. However, this approach is uncomfortable and prohibits continuous BP measuring due to physical constraints. The measurement result of this method is the most accurate, but it is uncomfortable and cannot measure continuous BP. Continuous blood pressure measurement can be achieved in an invasive (intra-arterial) way. However, it is an expensive and invasive procedure and carries an increased risk of complications [7]. Recently, BP estimation methods based on PPG have been widely studied. This method is noninvasive, simple and easy to implement [8].

Photoplethysmography (PPG) is a simple and low-cost optical technique that can be used to detect blood volume changes in the microvascular bed of tissue [9]. Currently, many smart wearable devices have built-in PPG sensors, which are widely used to measure heart rate (HR), heart rate variability (HRV), and oxygen saturation (SpO2) [10]. PPG waveform reflects the change of blood volume at the measurement position, which is closely related to the change of BP. Therefore, PPG can be used as a potential method to monitor continuous BP.

There are two kinds of approaches for estimating BP based on PPG, using either the PPG signal only or PPG signal along with other signals (e.g., electrocardiogram) [11]. In [12,13,14,15], Pulse Transit Time (PTT)-based methods are carried out. PTT is the time interval between the R-peak of an electrocardiogram (ECG) and the point with maximum gradient on the rising edge of the PPG [16]. Thus, it requires ECG and PPG measurements simultaneously, but it is difficult to ensure the synchronization of the signal, because the signal processing time of each device is different. When using ECG signal and PPG signal to obtain pulse arrival time (PAT) or PTT, their calculation methods are similar [17]. BP knowingly correlates with the pulse wave velocity (PWV) [11], thus many studies [18,19,20,21] use PWV as the feature parameter to estimate BP. The PWV requires not only calculating PTT or PAT but also measuring the distance between the heart and the index finger, which differs from one person to another [8].

Regarding the algorithms mentioned above, although the results obtained are satisfactory, two signals of the ECG signal and PPG signal are needed, and some algorithms need to obtain ECG and PPG signals synchronously, which is not convenient enough. Considering that the computational burden is less, it is more convenient to estimate BP with only one signal. Therefore, many researchers try to estimate BP using only PPG signals.

In [8,22,23,24], machine learning (ML) algorithms have been used to estimate BP from a PPG signal. In these studies, it is necessary to use the features of the ppg signal as the input of the ML model to estimate DBP and SBP. However, motion artifacts are often found diminishing the signal quality, which causes feature extraction failure [11]. In order to avoid the influence of feature extraction failure, some studies [11,25,26] try to estimate BP using the raw signal. In [25], the first and second derivatives of the PPG signal are used as the input of a modified ResNet-GRU-based network to estimate DBP and SBP. However, the ResNet-GRU-based model is computationally expensive as the learning efficiency of gated recurrent unit (GRU) is low and converges slowly [27]. In order to overcome this shortcoming, Harfiya [11] replaced the ResNet-GRU-based network with an LSTM-based network. However, these two algorithms need to obtain two derivatives of the raw signal; hence, the computational burden is relatively large. In addition, the ABP-Net is proposed based on fully convolutional neural networks (CNN) in [28]; ABP-Net has good performance in predicting the BP waveform, but it still needs the first and second derivatives of the raw PPG signal as input. Another research work [26], used two CNN to extract morphological features from each PPG segment and then estimated SBP and DBP separately. However, the model consists of multiple networks, which is computationally expensive.

In this study, we propose a model based on a generalized regression neural network (GRNN) to estimate the BP waveform (continuous BP) from the raw PPG signal and extract DBP and SBP from the estimated BP waveform. The model consists of an encoder, GRNN network and decoder. The encoder is responsible for decomposing the PPG signal into N harmonics. The GRNN takes the N harmonics of the PPG signal as input and outputs the N harmonics of the BP signal. Finally, the decoder is responsible for converting the output of GRNN into a time-domain waveform of BP. Our model can not only predict the BP waveform but also provide a frequency domain feature of the BP waveform. The frequency domain feature of the BP waveform is also required by some researches. For example, Zhang [29] used the N harmonics of the BP waveform to study the propagation and reflection of the pulse wave; Li and Wei [30] used the N harmonics of the peripheral BP waveform to study the diagnosis of arterial stenosis; Arvanaghi et al. [31] used arterial BP based on discrete wavelet transform to study the classification of cardiac arrhythmias.

The advantages of this study include:

1.  Estimating continuous and noninvasive BP waveforms directly from the raw PPG signal only, and there is no need for the first and second derivatives of the PPG signal;
2.  The input of the GRNN net is the amplitude and phase angle of the PPG signal in a specific frequency, no PPG signal features are required, and the model has a low computational burden;
3.  Our method can not only estimate DBP and SBP but also estimate the BP waveform and frequency domain feature of the BP waveform.

Our article is organized in the following manner. Section 2 explains the data sources, data preprocessing, model composition, and experimental settings. In Section 3, the experimental results are discussed and the different methods are compared. Section 4 discusses and summarizes the experimental results, and Section 5 concludes this study.

## 2. Materials and Methods

The physiological signal data used in this article comes from the Multiparameter Intelligent Monitoring in Intensive Care II (MIMIC II) online database [32] provided by the PhysioNet organization. The database can provide invasive Intra-Arterial BP signals and PPG signals collected from fingertips. There are 12,000 subjects in the database, and each instance consists of a PPG signal and a synchronously measured BP signal. The sampling frequency of both signals is 125 Hz. It should be noted that this database obtains data from the intensive care unit (ICU), which may contain abnormal BP signals due to the influence of drugs [33]. Therefore, it is necessary to preprocess the data in the database. After data processing, 3183 subjects were reserved for further experiments. In addition, since each record has a varying record duration, only the first 1000 samples of each instance are kept, as some of the records in the database possess a maximum of 1000 samples.

### 2.1. Data Preprocessing

#### 2.1.1. Wave Filtering

There is high-frequency and low-frequency noise in the PPG signals, so the first step of signal preprocessing is filtering. According to previous studies [11,20], the third-order Bass bandpass filter is used, and the range of the passband is from 0.5 to 8 Hz.

#### 2.1.2. Abnormal Signal Elimination

**Figure 1:**
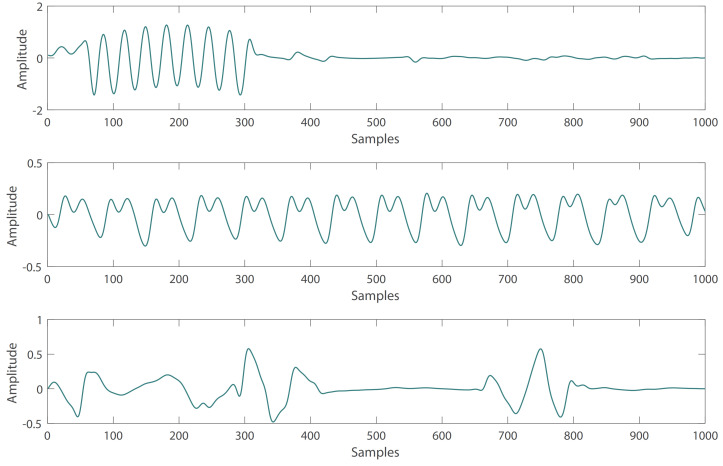
Abnormal PPG signals with irregular waveform.

**Figure 2:**
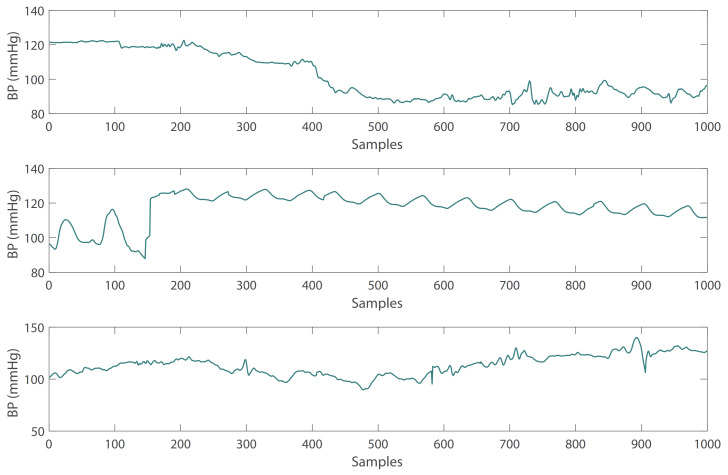
Abnormal BP signals with irregular waveform.

- The subjects with very high BP or very low BP were removed. To ensure that SBP is less than 180 and more than 80, DBP is less than 130 and more than 60.
- Affected by changes in sensor position or movement, some PPG waveforms are irregular in the subjects. These abnormal PPG signals can be removed by the automatic detection algorithm of the PPG systolic peak in the heartpy toolkit [34]. Some examples of irregular PPG waveforms are shown in Figure 1.
- Due to the influence of drugs, sensor movement and other factors, there are some abnormal BP waveform signals in the subjects. These abnormal signals can also be removed with the heartpy toolkit [34]. Some examples of abnormal BP waveforms are shown in Figure 2.

#### 2.1.3. Single-Period Waveform Extraction

**Figure 3:**
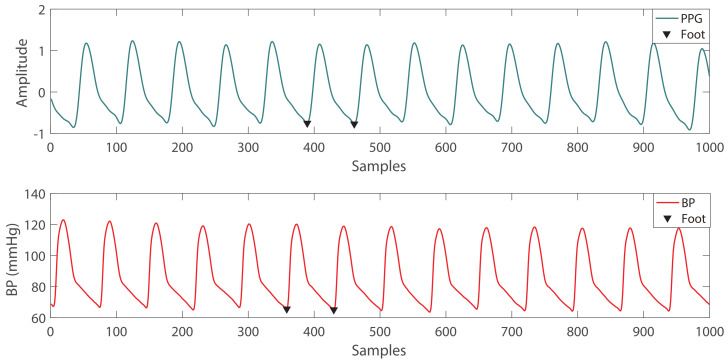
Example of single-period waveform extraction: the waveform between the two feet is extracted.

In order to obtain the amplitude and phase angle of the signal conveniently, it is necessary to extract the single-period PPG signal and BP signal from the periodic signal. An example of a single-period waveform extraction is shown in Figure 3, and the waveform between the two Feet is extracted. In addition, it is necessary to perform a Pearson’s correlation detection on the extracted single-period PPG and BP signals to determine the degree of similarity between PPG and BP signals in terms of morphology. Signals with an average Pearson’s correlation coefficient *r* less than 0.8 are removed. The Pearson’s correlation coefficient *r* is calculated as follows [35]:

(1) $$r=\frac{n\sum PB-\sum P\sum B}{\sqrt{n\sum P^{2}-{\left(\sum P\right)}^{2}}-\sqrt{n\sum B^{2}-{\left(\sum B\right)}^{2}}}$$

where *P* is the single-period PPG signal and *B* is the single-period signal.

**Figure 4:**
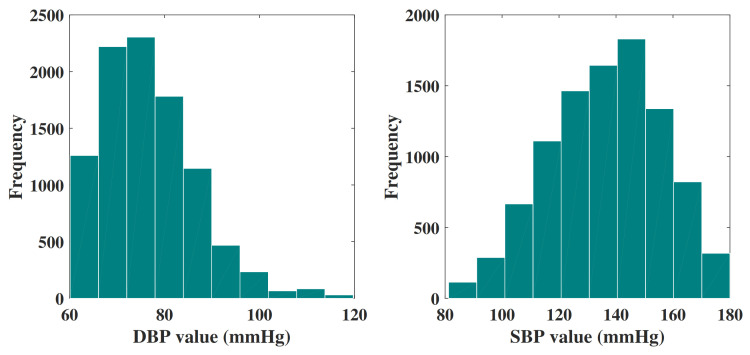
Histogram of DBP values and SBP values in the final data set.

The final data set consists of 9549 signal groups, and each of them is attributed a unique ID. In addition, each group of signals includes a PPG signal and a corresponding BP signal. All these signals are extracted from 3183 subjects. Then, the final data set is randomly divided into three groups: 75% for training, 15% for verification, and 15% for testing. Figure 4 shows the histogram distribution of the DBP values and SBP values in the final data set.

### 2.2. GRNN-Based Model

The model based on GRNN consists of an encoder, GRNN network and decoder. The encoder is responsible for encoding the single-period PPG signal into the N harmonics. The N harmonic of the PPG is used as the input of GRNN to estimate the N harmonics of the BP signal. The decoder is responsible for encoding the N harmonics of the BP signal into the time domain waveform of BP.

#### 2.2.1. Encoder and Decoder

As is known to all, if any periodic signal satisfies the Dirichri conditions, this periodic signal can be expanded into a Fourier series. Obviously, the PPG signal (p(t)) is a periodic signal that satisfies Dirichri conditions, so it can be expanded into a Fourier series:

(2) $$P\left(t\right)=P_{0}+\sum_{n=1}^{N}\left(P_{n}cos\left(n\omega_{0}t\right)+\varphi_{n}\right)$$

where $P_{0}$ is the DC component. $\omega_{0}=\frac{2\pi }{T}$ is the angular frequency of the fundamental frequency, *T* is the period of the PPG signal. *N* is the number of harmonics. $P_{n}$ is the amplitude of the nth harmonic. $\varphi_{n}$ is the phase angle of the nth harmonic.

After bandpass filtering, the frequency range of the PPG signal is [0.5,8]. This means that the PPG signal does not contain a DC component. Let N=9, then Equation (2) can be rewritten as:

(3) $$P\left(t\right)=\sum_{n=1}^{9}\left(P_{n}cos\left(n\omega_{0}t\right)+\varphi_{n}\right)$$

Therefore, the single-period PPG signal can be encoded as an array $P_{encoder}$:

(4) $$P_{encoder}=\left[P_{1},P_{2},\cdots ,P_{9},\varphi_{1},\varphi_{2},\cdots ,\varphi_{9}\right]$$

Similarly, the BP signal B(t) can also be expanded into series:

(5) $$B\left(t\right)=B_{0}+\sum_{n=1}^{N}\left(B_{n}cos\left(n\omega_{0}^{\left(B\right)}t\right)+\phi_{n}\right)$$

where $B_{0}$ is the DC component. $\omega_{0}^{\left(B\right)}=\frac{2\pi }{T^{\left(B\right)}}$ is the angular frequency of the fundamental frequency, $T^{\left(B\right)}$ is the period of the BP signal. N=17 is the number of harmonics. Here, the value of *N* is consistent with the prior study [29], so that the predicted BP waveform of our model can be used for more research. $B_{n}$ is the amplitude of the nth harmonic, $\phi_{n}$ is the phase angle of the nth harmonic. Then, the single-period BP signal can be encoded as an array $B_{encoder}$:

(6) $$B_{encoder}=\left[B_{1},B_{2},\cdots ,B_{17},\phi_{1},\phi_{2},\cdots ,\phi_{17}\right]$$

The parameters $P_{encoder}$ and $B_{encoder}$ can be obtained by the least squares curve fitting.

The least squares method is a mathematical optimization technique that finds the best function match of the data by minimizing the sum of squares of errors. The criterion for selecting the best-fitting curve can be determined to minimize the total fitting error (i.e., the total residual).

To determine the parameter $P_{encoder}$ as an example, there is a set of data $\left(t_{i},P\left(t_{i}\right)\right)$, and it is known in advance that they should satisfy a certain functional relationship $f(t_{i},P\left(t_{i}\right))$, such as Equation (3). Based on this known information, some parameters ($P_{encoder}$) need to be determined. Then, the goal is to find a set of $P_{encoder}$ that minimizes the value of the following function *S*:

(7) $$S\left(P_{encoder}\right)=\sum_{i=1}^{m}{\left(P\left(t_{i}\right)-f\left(t_{i},P\left(t_{i}\right)\right)\right)}^{2}$$

When the error is the smallest, the coefficient at this time is the best fitting state.

Only BP signals need to decode, since $B_{encoder}$ is the output of GRNN. The BP waveforms can be obtained by taking $B_{encoder}$ into Equation (5).

#### 2.2.2. GRNN

GRNN was proposed by D.F.Specht in 1991 [36], and it is a modified form of a radial basis network (RBF). GRNN is based on non-parametric regression, using sample data as a posterior condition, performing Parzen non-parametric estimation, and calculating the network output according to the principle of maximum probability. GRNN is based on RBF, so it has good nonlinear approximation performance. Therefore, GRNN is very suitable for approximation from $P_{encoder}$ to $B_{encoder}$. The difference between GRNN and RBF is that there is an extra layer of summation, and the weight connection between the hidden layer and the output layer is removed.

**Figure 5:**
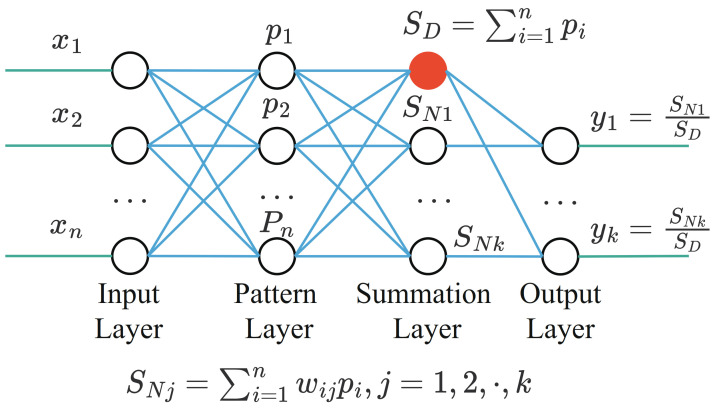
The GRNN structure.

The GRNN structure diagram is shown in Figure 5. It consists of:

1.  The input layer, which is fully connected with the pattern layer. The number of nodes is equal to the feature dimension of the sample;
2.  The pattern layer, the number of nodes is equal to the number of training samples, the pattern function can be calculated as:
  (8) $$p_{i}=exp\left[-\frac{{\left(x-\mu_{i}\right)}^{T}\left(x-\mu_{i}\right)}{2\delta^{2}}\right]$$
  where *x* is input vector, $\mu_{i}$ is the training vector corresponding to the i-th neuron, δ is a hyperparameter of the model and needs to be set in advance.
3.  The summation layer, the number of nodes is one more than the output sample dimension. The output of the summation layer is divided into two parts. The output of the first node is the arithmetic sum $S_{D}$ of the output of the mode layer, and the output of the remaining nodes is the weighted sum $S_{Nj}$ of the output of the mode layer.
4.  The output layer, the number of nodes in the output layer is equal to the dimension of the output vector. The output of each node is equal to the output of the corresponding summation layer divided by the output of the first node of the summation layer.

In our model, the input vector $x=P_{encoder}$, the output vector $y=B_{encoder}$, *n* is the number of training samples and k=17. Then we will take the calculation process of $B_{1}$ (the first element in $B_{encoder}$) as an example to explain how to obtain $B_{encoder}$ from $P_{encoder}$. The first step is to calculate the transfer function $tf_{i}$ of the pattern layer:

(9) $$tf_{i}=exp\left[-\frac{{\left(P_{encoder}^{\left(B_{1}\right)}-P_{encoder}^{i}\right)}^{T}\left(P_{encoder}^{\left(B_{1}\right)}-P_{encoder}^{i}\right)}{2\delta^{2}}\right],\;i=1,2,\cdots ,n$$

where $P_{encoder}^{i}$ is the $P_{encoder}$ corresponding to the ith training sample, and $P_{encoder}^{\left(B_{1}\right)}$ is the $P_{encoder}$ corresponding to the test sample where $B_{1}$ is located

The second step needs to solve $S_{D}$ and $S_{N1}$:

(10) $$S_{D}=\sum_{i=1}^{n}P_{i}=\sum_{i=1}^{n}exp\left[-\frac{{\left(P_{encoder}^{\left(B_{1}\right)}-P_{encoder}^{i}\right)}^{T}\left(P_{encoder}^{\left(B_{1}\right)}-P_{encoder}^{i}\right)}{2\delta^{2}}\right]$$

(11) $$S_{N1}=\sum_{i=1}^{n}\omega_{i1}P_{i}=\sum_{i=1}^{n}\omega_{i1}exp\left[-\frac{{\left(P_{encoder}^{\left(B_{1}\right)}-P_{encoder}^{i}\right)}^{T}\left(P_{encoder}^{\left(B_{1}\right)}-P_{encoder}^{i}\right)}{2\delta^{2}}\right]$$

where $\omega_{i1}$ the first element in $B_{encoder}$ corresponding to the ith training sample.

Finally, $B_{1}$ can be obtained from:

(12) $$B_{1}=\frac{S_{N1}}{S_{D}}$$

#### 2.2.3. Model Setup

**Figure 6:**
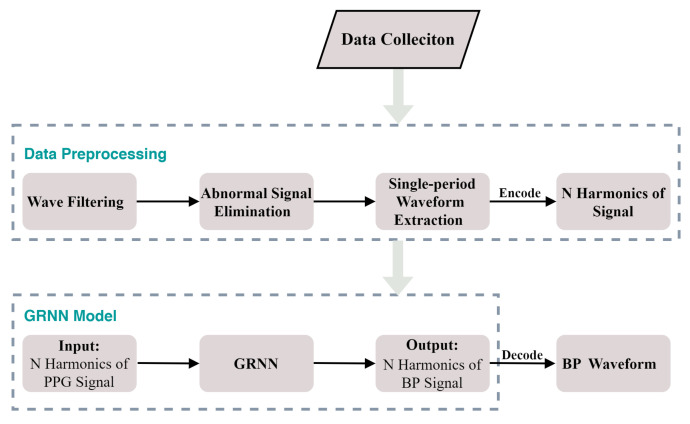
The block diagram of our proposed model.

In our proposed model, the parameter δ of GRNN is set to 0.001. Our GRNN model was generated by the MATLAB${}^{®}$ software toolbox R2017b. The block diagram of our proposed model is shown in Figure 6. In our experiment, a desktop computer with intel core i7-10700k @3.8 GHz, 32 GB RAM and NVIDIA GTX 2080 Ti 11 GB graphics card was used. The average time to obtain each BP waveform was about 0.4 s.

## 3. Results

The results of the model are evaluated by the mean absolute error (MAE) and the root mean square error (RMSE), which are calculated in Equations (13) and (14), respectively.

(13) $$MAE=\frac{1}{N}\sum_{i=1}^{N}|d_{i}|$$

(14) $$RMSE=\sqrt{\frac{1}{N}\sum_{i=1}^{N}d_{i}^{2}}$$

where *d* is the error, which is the difference between the model output and the actual value. *N* is the number of samples.

In Table 1, all the studies use PPG and BP signals from the same database (MIMIC II). However, some models (SVR, ERM and GDNN) need to extract features of the PPG signal as input, while other methods do not. Reference [37] proposed 14 new features based on the five characteristic points of the second derivative of the PPG signal and combined the new features with the conventional 21 time-scale PPG features for training an SVR. The reported result shows 40% accuracy improvement as compared with a conventional 21-feature based neural network method. Reference [22] selects 59 features as the input of GDNN to estimate SBP and DBP. Among all the models listed in the table, this model achieves the best results, and its SBP and DBP prediction errors are the smallest. However, it cannot estimate the BP waveform, which limits the application of this model. Reference [8] proposed a 7-feature based enhanced regression model. Although this model requires fewer features, the DBP prediction error is larger. In [38], an end-to-end deep learning algorithm with an attention mechanism was proposed to estimate BP. The method does not require a feature extraction process, but the accuracy of this method is worse than the method proposed in Reference [39]. Reference [39] uses the raw PPG signal as the input of CNN to estimate the ABP waveform and obtain a smaller MAE. However, their RSME is higher than other models that use the raw PPG signal. This indicates that the prediction error dispersion of the model is relatively high. Reference [11] developed an LSTM-based autoencoder model to estimate the whole waveform of BP. The input to the model is the PPG signal and its first and second derivatives. Their RMSE is better than ours, but MAE is higher. Moreover, our model is simpler to construct and has less computational burden, and our model can also provide the frequency domain characteristics of the BP signal.

The comparison of our proposed model results with the British Hypertension Society (BHS) Standard is shown in Table 2. This standard grades the BP measurement system, based on the cumulative error, to be less than their three different thresholds (5, 10, and 15 mmHg) [40]. According to this standard, the BP estimation from our proposed GRNN-based model is obviously consistent with the grade A for both SBP and DBP.

The comparison of our proposed model results with the Association for the Advancement of Medical Instrumentation (AAMI) Standard [41] is shown in Table 3. The standard stipulates that the average prediction result error and standard deviation error (STD) of 85 subjects must be lower than 5 and 8 mmHg, respectively. The prediction results of our model meet all the above criteria. The MAE and STD for predicting SBP are 3.96 and 5.36, respectively, and the MAE and STD for predicting DBP are 2.39 and 3.28, respectively.

## 4. Discussion

**Figure 7:**
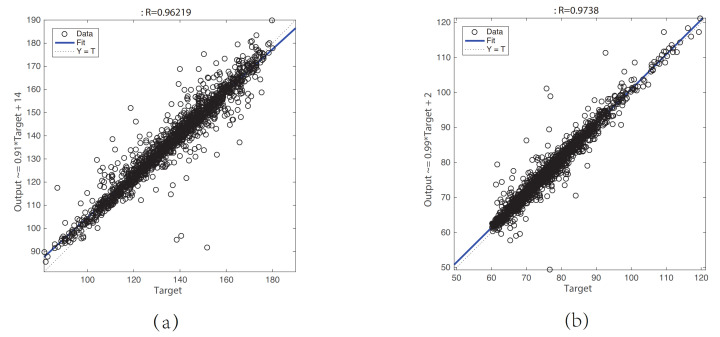
Linear regression plot of the (**a**) SBP and (**b**) DBP result.

A PPG signal is widely used to obtain the information of cardiovascular systems and respiratory systems because of its noninvasive and versatility [11]. In recent years, it has become a trend to use only PPG signals to estimate BP signals. For non-ideal PPG signals, it is impractical to extract the time-domain waveform characteristics. Therefore, we propose a GRNN model for estimating the BP waveform with the raw PPG signal as input. Because both PPG signals and BP signals can be regarded as a superposition of N harmonics, we encode the PPG signal and the BP signal into the amplitude and phase angle ($P_{encoder}$ and $B_{encoder}$) of the N harmonics using the least squares method. In this way, the mapping problem between the PPG waveform and BP waveform can be converted into a mapping problem between $P_{encoder}$ and $B_{encoder}$. This greatly reduces the difficulty of modeling machine learning models. Linear regression plots of the (a) SBP and (b) DBP results are shown in Figure 7. The results show that the correlation coefficient R=0.96 between the target SBP and the predicted SBP, the equation of the linear fitting:

(15) $$SBP_{prediction}\;=0.91*SBP_{target}+14$$

where $SBP_{prediction}$ is the predicted SBP value of the model, and $SBP_{target}$ is the target SBP value. The correlation coefficient R=0.97 between the target DBP and the predicted DBP, the equation of the linear fitting:

(16) $$DBP_{prediction}\;=0.99*DBP_{target}+2$$

where $DBP_{prediction}$ is the predicted DBP value of the model, and $DBP_{target}$ is the target DBP value, which indicates that the prediction results are basically accurate except for a few cases.

**Figure 8:**
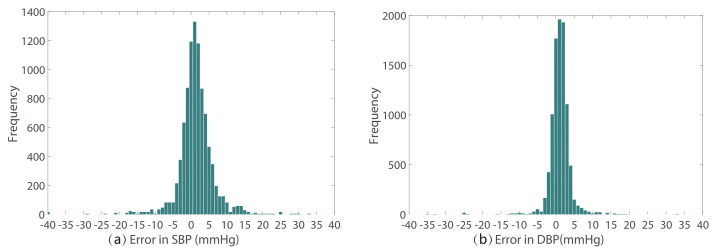
The prediction error of (**a**) SBP and (**b**) DBP.

**Figure 9:**
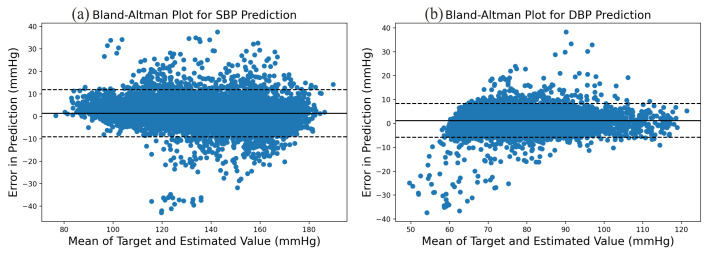
Bland–Altman Plot for (**a**) SBP prediction and (**b**) DBP prediction.

The prediction error statistics of SBP and DBP are shown in Figure 8. It can be seen from the figure that the error distribution is around the zero value and basically conforms to the normal distribution. Two Bland–Altman plots have been reported in order to test the consistency of the estimated value with the target value. The Bland–Altman plot for SBP Prediction and DBP Prediction is shown in Figure 9. The 95% limits of consistency span the segment from μ−1.96δ to μ+1.96δ (shown using dashed lines), where μ and δ are the mean and standard deviation of the prediction error, respectively. For SBP and DBP, this limit translates to [−9.10,11.92] and [−5.28,7.58] mmHg, respectively. It can be seen from the Bland–Altman plots that most of the prediction errors are within the consistency limit, and the distribution of the errors outside the consistency limit conforms to the BHS standard (Table 2) and AAMI standard (Table 3). Therefore, it can be considered that the prediction values are in good consistency with the target value.

Compared with other models listed in Table 1, the prediction results of our proposed model are not the best. However, compared with the feature-based model, our model has the advantage of being able to estimate the BP waveform.

**Figure 10:**
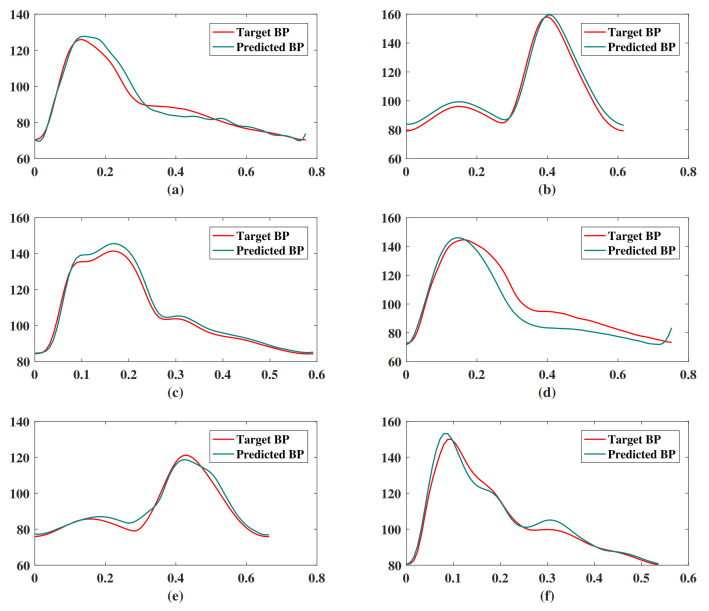
Examples of BP waveform prediction results of the proposed model. These subgraphs are randomly selected targets and predicted BP waveforms of (**a**) segment ID 287, (**b**) segment ID 1199, (**c**) segment ID 4764, (**d**) segment ID 8778, (**e**) segment ID 2372, (**f**) segment ID 91.

**Figure 11:**
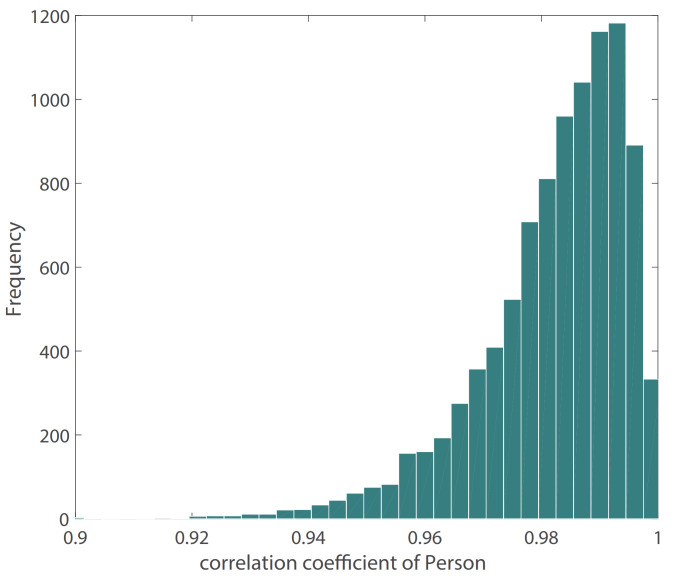
Distribution of Pearson’s correlation coefficient between target and estimated BP waveform.

Figure 10 shows the result of our BP waveform prediction using the proposed model, which has a high similarity with the target waveform from the source data set. It shows that the proposed model not only predicts SBP and DBP accurately but also predicts the BP waveform accurately. Since the Pearson’s correlation coefficient can be used to measure the similarity between two time series data [39], we estimated the Pearson’s correlation coefficient (*r*) between the predicted and target BP waveforms in order to evaluate the prediction results of the BP waveform. The distribution of *r* has been shown in Figure 11. The figure shows that most of the *r* values are in [0.9,1], which indicates that most of the predicted BP waveforms have a high correlation with the target BP waveforms. This *r* is also estimated in the [39], and Table 4 lists our and their results. The comparison results show that the performance of our model is close to that of the CNN model [39]. The value of the 25th and 75th percentile of *r* indicates that our model predicted most of the waveform accurately.

The estimated waveform contains more physiological information than SBP and DBP, which is helpful for the diagnosis of cardiovascular diseases. Compared with other models that can estimate the BP waveform, our MAE is better than Reference [11], and our RSME is better than Reference [39]. In addition, our model is easier to build and has a lower cost.

However, about 75% of the data in the database is removed in the data preprocessing stage. As a result, the amount of data used is less and the breadth is insufficient, which may lead to poor robustness of the proposed model. Based on this, future studies should focus on finding additional data sources and develop more complex models to adapt to abnormal signals.

## 5. Conclusions

A model based on GRNN is proposed, which uses the PPG signal to estimate the BP waveform. Considering that the PPG signal feature may fail to be extracted, our model uses the raw PPG signal as input. The predicted BP waveform of the model is highly correlated with the target BP waveform. Moreover, the model can also provide a fairly accurate estimation result of SBP and DBP, which can meet the requirements of the AAMI standard. According to the BHS standard, the SBP and DBP estimation results of our model both achieve Grade A. In the future, the studies should focus on finding additional data sources and develop more complex models to adapt to abnormal signals.

## Figures and Tables

**Table 1:** Performance comparison between different BP estimation models.

Model	SBP(mmHg)		DBP(mmHg)	
	MAE	RMSE	MAE	RMSE
Support Vector Regression (SVR) [37]	8.54	10.9	4.34	5.8
Generalized Deep Neural Network (GDNN) [22]	3.21	4.63	2.23	3.21
Enhanced regression model (ERM) [8]	4.24	5.06	4.81	6.37
Long Short-Term Memory (LSTM) [11]	4.05	5.25	2.41	3.17
End-To-End Deep Learning Architecture(ETE) [38]	4.06	5.42	3.33	4.30
Convolutional neural network (CNN) [39]	3.68	5.75	1.97	3.52
Our model	3.96	5.54	2.39	3.45

**Table 2:** Comparison result with BHS Standard.

Cumulative Error Percentage				
**Error**		**≤5 mmHg**	**≤10 mmHg**	**≤15 mmHg**
Our result	SBP	80.1%	93.9%	97.6%
	DBP	93.9%	98.1%	99.2%
BHS	Grade A	60%	85%	95%
	Grade B	50%	75%	90%
	Grade C	40%	65%	85%

**Table 3:** Comparison result with AAMI Standard.

		MAE	STD	Subjects
Our result	SBP	3.96	5.36	3183
	DBP	2.39	3.28	3183
AAMI		<5	<8	>85

**Table 4:** Comparison of BP waveform prediction performance between our model and CNN model.

Evaluation Factor	CNN Model [39]	Our Model
Average *r*	0.993	0.981
Minimum *r*	0.262	0.321
Maximum *r*	0.999	0.999
25th percentile of *r*	0.989	0.976
75th percentile of *r*	0.996	0.992

## Data Availability

A publicly available dataset (MIMIC II) was used in this study, which can be found here: https://archive.ics.uci.edu/ml/datasets/Cuff-Less+Blood+Pressure+Estimation (accessed on 25 July 2021).
